# Supplementary material for: Predicting Bacteremia among Septic Patients Based on ED Information by Machine Learning Methods: A Comparative Study
Source: Diagnostics (Basel). 2022 Oct 15;12(10):2498. doi: 10.3390/diagnostics12102498 (PMC9600599; doi:10.3390/diagnostics12102498)
Supplement: Supplementary file 1 [file diagnostics-12-02498-s001.zip › diagnostics-1875697-supplementary.pdf]

Supplementary Table S1. Definition of comorbidity.

	Corresponding ICD-10 codes
Myocardial infarction	I21;I22;I252;I255
Congestive heart failure	I110;I130;I132;I50
Peripheral vascular disease	I70;I71;I731;I738;I739;I771;I790;I792;K551; K558;K559; Z958;Z959
Old stroke or TIA	G45;G46;H340;I60-I69
Dementia	F00-F03;F051;G30;G311
COPD	I278;I279;J40-J47;J60-J67;J684;J701;J703
Connective tissue disease	M05;M06;M315;M32;M33;M34;M351;M353;M360
Peptic ulcer disease	K25-K28
Mild liver disease	B18;K700-K703;K709;K713K715;K717;K73; K74;K760; K762-K764;K768;K769;Z944
Uncomplicated diabetes	E100;E101;E106;E108;E109;E110;E111;E116; E118;E119; E120;E121; E126;E128-E131;E136; E138-E141; E146;E148; E149
Moderate to severe CKD	I120;I131;N032-N037;N052-N057;N18;N19; N250;Z490;Z491; Z492;Z940;Z992
Hemato-oncology	C00-C26;C30-C34;C37-C41;C43; C45-C58; C60-C76;C81-C85;C88; C90-C97
Metastatic solid tumor	C77-C80
HIV infections	B20-B22;B24;Z21

The patient's medical history was retrieved from the electronic medical record with ICD-10 codes corresponding to these diseases.

ICD = international classification of diseases; CKD = chronic kidney disease; COPD = chronic obstructive pulmonary disease; HIV = human immunodeficiency virus; TIA = transient ischemic accident.
